# Asymptomatic Evolution and Regression of Temporal Lobe Necrosis After Adjuvant Radiation for Skin Cancer: A Case Report and Review of Literature

**DOI:** 10.7759/cureus.481

**Published:** 2016-02-05

**Authors:** Rima Pathak, Gaurav Bahl

**Affiliations:** 1 Radiation Oncology, University of British Columbia, BC Cancer Agency; 2 Division of Radiation Oncology and Developmental Radiotherapeutics, University of British Columbia, BC Cancer Agency

**Keywords:** temporal lobe necrosis, radiation therapy, skin cancer

## Abstract

Temporal Lobe Necrosis (TLN) is not an expected complication of adjuvant radiation therapy (RT) for skin cancers and has become uncommon otherwise in daily practice due to improved RT planning and modern delivery techniques. TLN is a great mimic and can be mistaken for disease recurrence, metastasis to the brain, or high grade primary brain tumor. This case report demonstrates the importance of diagnosing the entity, its natural evolution, and dosimetric correlation with published constraints.​ It emphasizes the importance of thorough clinical examination on follow-up and review of previous radiation plan when encountered with challenging differentials. We also provide a review of clinical presentations, imaging modalities, and management options for patients with suspected TLN.

## Introduction

Radiation-induced temporal lobe necrosis (TLN) is an uncommon side effect usually associated with high doses of radiation for nasopharyngeal carcinoma (NPC), pituitary adenoma, and skull base tumors. It is however, not an expected complication of adjuvant radiation therapy (RT) for skin cancers. TLN has become uncommon in daily practice due to improved RT planning and modern delivery techniques.

The incidence of TLN varies from 0%–24% with conventional fractionation to 35% with accelerated fractionation [1-3]. Brain tissue is known to be highly vulnerable to changes in dose fractionation and treatment times. The symptoms range from being asymptomatic to severe morbidity and frequently mortality. TLN is a great mimic and can be mistaken for disease recurrence, metastasis to the brain, or a high grade primary brain tumor. This case report demonstrates the importance of diagnosing the entity, its natural evolution and dosimetric correlation with published guidelines. Informed patient consent was obtained for this study.

## Case presentation

A 74-year-old male patient presented with a 1.5 cm ulcerated swelling over the skin of his left pinna in October 2008. Biopsy revealed an invasive moderately differentiated squamous cell carcinoma (MDSCC) which was excised with wide margins. In April 2009, he developed recurrent disease posterior to the angle of the mandible with regional metastasis to the parotid and suspicious involvement of ipsilateral level II neck nodes. A total parotidectomy with modified radical neck dissection was performed in May 2009. Pathology confirmed MDSCC in the parotid with positive margins. All dissected lymph nodes were negative. He received a hypofractionated course of adjuvant RT to the parotid region: 60 Gy/25 fractions (2.4 Gy per fraction, one fraction per day, five days per week). RT was planned with 6 MV photons using a 5-field 3D conformal technique. He tolerated treatment reasonably well and completed treatment in September 2009 without interruptions.

**Figure 1:**
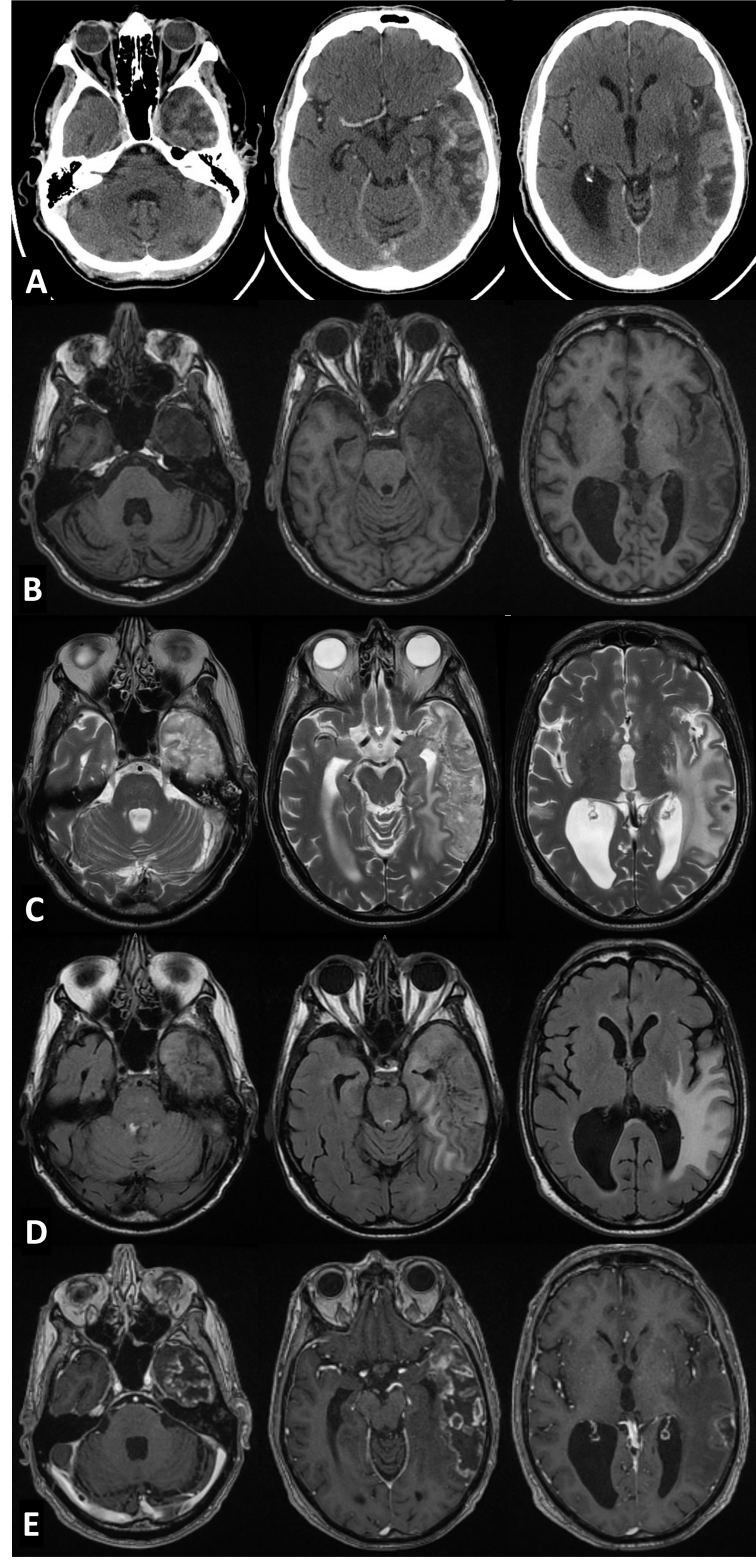
Incidental imaging findings of the patient A- Enhancing lesion with central necrosis on contrast-enhanced CT scan. B- Hypo-intense lesion with effacement of temporal horn of the lateral ventricle on left side on T1 W images. C- Hyper-intense lesion with areas of cystic degeneration T2 W images. D- Extent of vasogenic edema seen on T2-FLAIR images. E- Ring enhancement with central necrosis on post contrast images.

At 18 months post-RT, a small patch of osteoradionecrosis was noted in the left external auditory canal. This was treated conservatively and it healed well. A follow-up head and neck computed tomography (CT) scan was done in June 2013 (Figure 1A), when his care was transferred to a new physician. This reported an incidental finding of an irregular enhancing focus involving the left temporal lobe and extending to left parietal lobe measuring about 8 x 3.3 cm in size. It was associated with moderate white matter edema extending up to the periventricular location of the posterior horn of the lateral ventricle. The differential diagnoses were brain metastasis and subacute cerebral infarct. There was no evidence of recurrence in the post-op or irradiated region. The patient denied development of any new symptoms. However, his wife had noted some short term memory loss and occasional word-finding difficulty over the last few months. A clinical examination revealed a stable lower motor neuron facial palsy, which he had developed postoperatively in 2009. Neurological examination was otherwise unremarkable.

A magnetic resonance imaging scan (MRI) of the brain (Figures 1B-1E) revealed a heterogeneous mass with peripheral nodular enhancement and some areas of T2-weighted-fluid-attenuated inversion recovery (T2/ FLAIR) signal hyperintensity. The surrounding vasogenic edema was seen reaching high parietal lobe superiorly and left hippocampus medially. 

**Figure 2:**
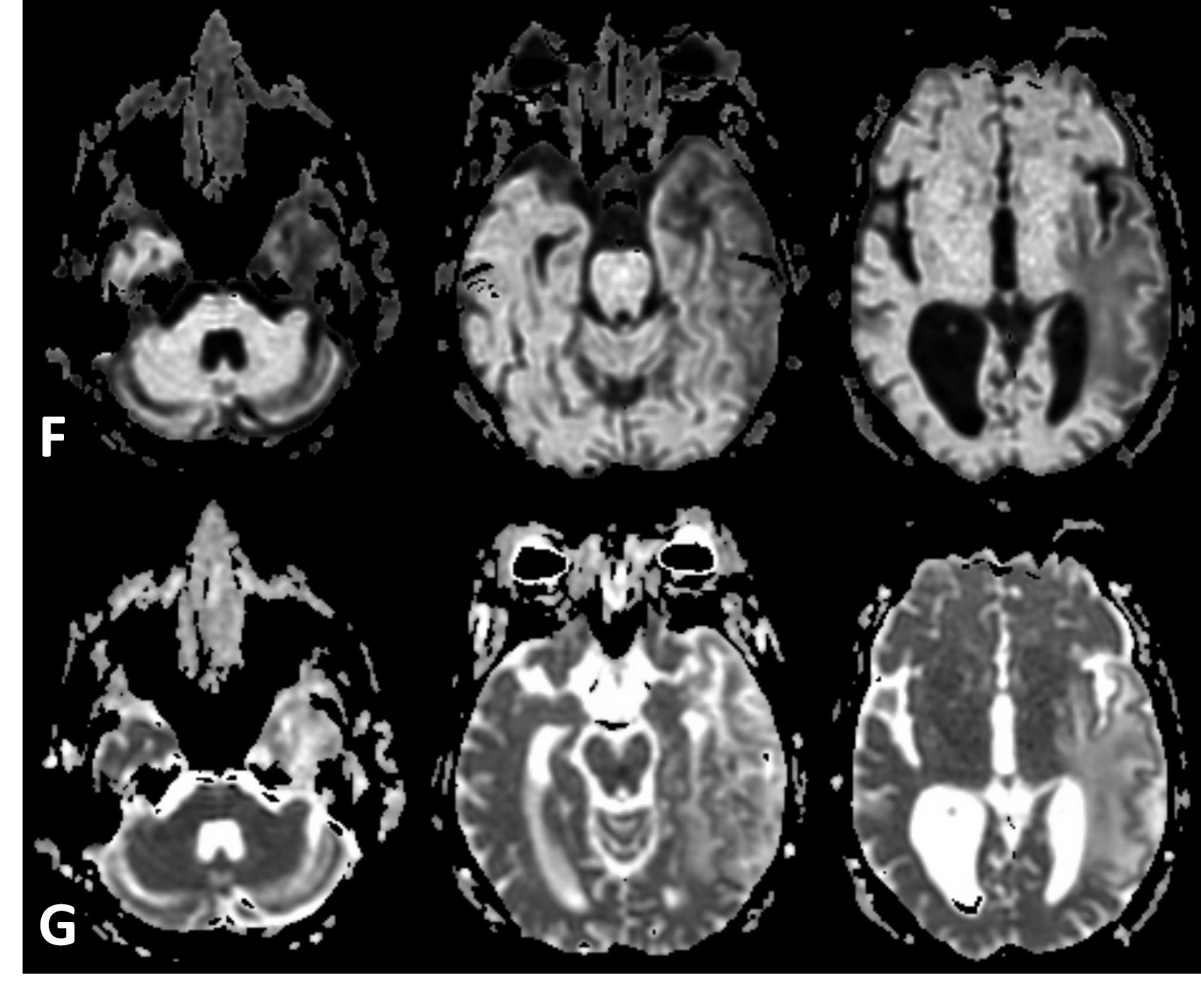
Diffusion weighted images for characterizing the lesion F- No abnormal restriction of fluid noted on exponential apparent diffusion coefficient images eliminating T2 shine-through effect. G- No abnormal restriction of fluid noted on apparent diffusion coefficient images.

There was no evidence of abnormal restricted diffusion (Figures 2F-2G). The differential diagnoses were revised to exclude subacute infarction and include radionecrosis or infection.

**Figure 3:**
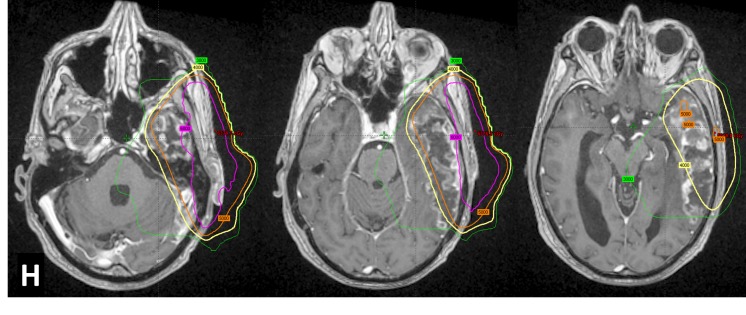
Super-imposition of treatment planning isodoses on the axial post contrast MRI images H- Extent of temporal lobe necrosis following the 40 Gy isodose (yellow), 60 Gy isodose confines to the extracranial tissue (pink) and the lesion mostly encompassed by the 50 Gy isodose (orange).

**Figure 4:**
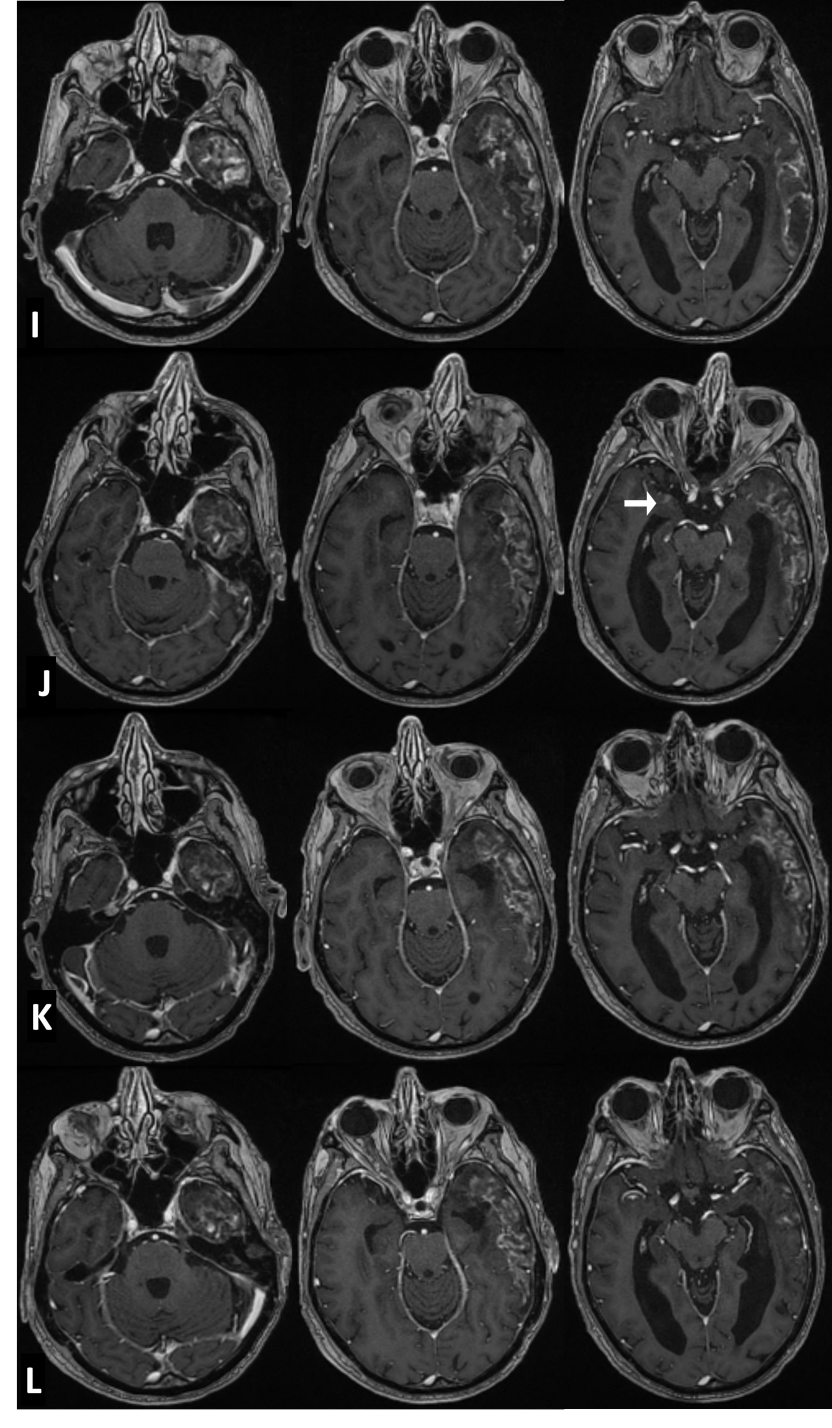
Follow-up axial post-contrast MRI images I- At three months, no change in enhancement. J- At nine months, development of new contralateral temporal lobe enhancing lesion (white arrow). K- At 18 months, mild temporal lobe atrophy with stable enhancement pattern in both the lesions. L- At 24 months encephalomalacia with ex-vacuo dilatation of the temporal horn and marked temporal lobe atrophy, resolution of contralateral enhancing lesion.

The radiation treatment plan was reviewed to rule out radionecrosis of the temporal lobe. The extent of the lesion in the temporo-parietal lobes correlated with the shape of the isodose curves on the treatment planning system, and hence, radionecrosis of the temporal lobe was established as the working diagnosis (Figure 3H). As the patient was asymptomatic, it was decided to observe the natural evolution of this lesion with serial imaging and intervene early if any new signs or symptoms developed. Subsequent MRI at three months (Figure 4I) showed reduction in edema and mass effect; however, the enhancement remained unchanged. At nine months, the MRI showed a new well-defined sub-centimeter enhancing nodule in the contralateral para hippocampal cortex reported as progression of metastatic disease (Figure 4J). A neuroradiology consultation suggested that the lesion could be a part of the evolving radionecrosis; however, there remained a distinct possibility of it being metastasis. In view of the lack of new clinical signs or symptoms, we decided to continue observation. An imaging at 12 months revealed no change in the size or characteristics of either lesion.

An MRI scan done in September 2014 reported that there was regression in the edema and T2/FLAIR changes but there was no change in the contralateral enhancing nodule (Figure 4K). An MRI scan at two years reported significant encephalomalacia with ex-vacuo dilatation of the temporal horn with loss of volume of the temporal lobe cortex and white matter (Figure 4L). The contralateral enhancing lesion had resolved. Throughout this follow-up period, the patient remained healthy and his clinical condition remained stable without any intervention. The patient remains well 30 months after the first suspicious CT. 

## Discussion

Temporal lobe necrosis is most commonly associated with radical RT for NPC and is rarely seen with other extracranial malignancies. Ironically, it was first described by Fisher and Holfelder after treatment for basal cell carcinoma of the temporal region in 1930 [4]. The temporal lobes have a variety of functions related to hearing, memory, visual perceptions, processing of semantics and complex inputs. Injury to one or both temporal lobes may present as problems with short-term memory, selective attention, recognizing faces, understanding spoken words or finding words (the only symptom in our patient). Severe injury to the amygdala in both the lobes could occur with treatment for skull base malignancies and may present with Kluver-Bucy Syndrome with symptoms like amnesia, hyperphagia, aggressive uninhibited sexual behaviour with persistent talking and visual agnosia. Vasogenic edema can result in symptoms of a mass effect, such as headaches, nausea, vomiting, photophobia, increased irritability, and diplopia. Necrosis and edema can lead to a hypercoagulable state resulting in thrombus formation and stroke. TLN has a variable latency period from three months to 13 years; however, most present within the first five years after completion of radiation therapy.

It is not uncommon to mistake TLN with a high-grade primary brain tumor, disease recurrence or brain metastasis. There are various imaging modalities that help in differentiating the above: MRI (diffusion-weighted imaging, spectroscopy, and perfusion), positron emission tomography (PET)-CT, and PET-MRI scan. Due to ease of availability, patients often first undergo a CT scan or an MRI. MRI features include white matter lesions that are predominantly hyperintense on T2-weighted images with thin-walled cystic areas. Vasogenic edema causes mass effect and gray matter changes like thinning of the cortex with irregular or blurred gray-white matter junction. A post-contrast scan shows typical swiss-cheese-, ring- or finger-like enhancement with surrounding hemosiderin deposition [5]. An MR spectroscopy usually reveals high lipid lactate peaks with low choline and N-acetyl aspartate peaks. The necrotic tissue shows hypoperfusion on dynamic susceptibility contrast-enhanced MRI. In conjunction with perfusion, the apparent diffusion coefficient maps (no restriction) help in the diagnosis. A PET-CT scan divides the radionecrosis into three different types depending on the stage of evolution: edema type, liquefactive necrosis type, and atrophic calcification type in that order [6]. For our patient, the strongest evidence came from the morphology and extent of the enhancing lesion and its correlation with the isodose distribution on the RT planning images. Hence, it was a valuable and inexpensive link to the diagnosis. As seen in Figure 3H, the enhancing and necrotic lesion follows the 40 Gy isodose line almost consistently. However, most of the affected brain tissue was enclosed by the 50 Gy isodose.

The treatment options for TLN include high doses of steroids in the early stages to control edema and mass effect; anticoagulants, antiplatelets, and vitamins like alpha-tocopherol to prevent thrombus formation and stroke [7]. Some reports indicate that hyperbaric oxygen initiates cellular and vascular repair and may be beneficial [8]. Recently, bevacizumab has been used in patients and has resulted in improvement in imaging characteristics and neurocognitive deficits. The initial response to bevacizumab can be dramatic and may be utilized for confirmation of diagnosis in the absence of histological evidence [8]. Surgery is indicated for TLN as a palliative measure to reduce symptoms of mass effect and has the advantage of providing histopathological confirmation of diagnosis.

About 65% of radiation-therapy-related deaths from NPC occur due to TLN [3]. Despite aggressive therapy, this condition can be fatal, and hence, the focus should be on prevention. Many studies have emphasized the critical importance of fraction size, overall treatment time, and product of total dose and fraction size [3]. Table 1 shows various dose constraints for effectively preventing TLN based on retrospective studies [1-3, 9-15]. These vary from a maximum allowable dose as low as 45 Gy to as high as 69 Gy. As fraction size has been found to be important, some recommendations are based on biological equivalent dose (BED) (with α/β ratio of two or three). Table 1 also compares the dosimetric parameters for our patient with recommended constraints from literature.

**Table 1:** Review of suggested dose constraints in literature, and comparison with dose received by our patient a: rV40, Percentage of Temporal Lobe Receiving ≥ 40 Gy, b: TLN, Temporal Lobe Necrosis, c: aV40, Absolute Volume of Temporal Lobe Receiving ≥ 40 Gy, d: BED, Biologically Equivalent Dose.

Authors	Number n	Tumor/Cancer Type	Suggested Constraints	End Point of Temporal Lobe Necrosis	For Prescribed Dose 60 Gy/25	Within Suggested Limit	Dose Actually Received by Temporal Lobe	Within Suggested Limit
Emami, et al. (1991) [9]	Review	Mixed	1/3^rd^ brain receiving 60 Gy	5% at 5 yrs	< 1% brain receiving 60 Gy	Yes	1/3rd brain received 11.5 Gy	Yes
Lawrence, et al. (2010) (QUANTEC) [10]	4675	Mixed	Fraction size < 2.5 Gy Dmax < 120 Gy_3 _(100- 140 Gy_3_)	5% overall	108 Gy_3_	Yes	83.3 Gy_3_	Yes
Lee, et al. (1998) [3]	1008	Nasopharyngeal Carcinoma	EQD2 ≤ 64	5% at 10 years	64.8 Gy	Almost	EQD2 50Gy	Yes
			Dmax < 104 Gy_3_	5% at 10 years	108 Gy_3_	Almost	Dmax 108 Gy_3_	Almost
Su, et al. (2013) [11]	870	Nasopharyngeal Carcinoma	rV40^a ^< 10%	TLN^b^ probability < 2.5%	rV40^a^ 49%	No	rV40^a^ 49%	No
			aV40^c ^< 5cc	aV40^c^ 43cc		No	aV40^c^ 43cc	No
Jeremy, et al. (2006) [2]	426	Primary Brain Tumor/Pituitary Adenoma	BED^d^ < 85.5 Gy_2_	6% overall	132 Gy_2_	No	BED^d^ 100Gy_2_	Yes
Marks, et al. (1981) [12]	152	Primary Brain Tumor/Pituitary Adenoma	Dmax ≥ 45 Gy	5%	Dmax 61 Gy	No	Dmax 61 Gy	No
			BED^d^ < 86.4 Gy_3_	TLN^b^ unlikely	108 Gy_3_	No	BED^d^ 83.3 Gy_3_	Yes
			Dmax ≥ 62.5Gy	25% at 5 yrs	Dmax 61 Gy	Yes	Dmax 61 Gy	Yes
Lee, et al. (2002) [1]	89	Nasopharyngeal Carcinoma	60 Gy/25# over 5 weeks	2.3 % at 5 yrs	64.8 Gy	Yes	50Gy/25# over 5 weeks	Yes
Sheline, et al. (1980) [13]	80	Mixed	Dmax ≤ 52Gy	TLN^b^ unlikely	Dmax 61 Gy	No	Dmax 61 Gy	No
Haberer, et al. (2010 )[14]	Review	Mixed	1/3^rd^ Brain receiving 60 Gy	5% at 5 yrs	<1% Brain receiving 60Gy	Yes	<1% Brain receiving 60Gy	Yes
Sun, et al. (2013) [15]	20	Nasopharyngeal Carcinoma	D_0.5cc _≤ 69 Gy	TLN^b^ unlikely	Dmax 61 Gy	Yes	Dmax 61 Gy	Yes

It was interesting to note that despite a prescribed dose to planning target volume (PTV) of 60 Gy/25, the dose received by the brain parenchyma was about 50 Gy/25 or less (Figure 3H). The contralateral temporal lobe received doses less than 10 Gy and yet showed subtle features of radionecrosis. On comparing dosimetric data of our patient (both ipsilateral and contralateral temporal lobes) with the recommendations made by various authors in Table 1, we understand that this patient probably had higher inherent radiosensitivity to experience temporal lobe necrosis at low doses that are considered safe in the absence of concurrent chemotherapy. Another explanation would be damage to the subventricular and subgranular zone stem cell niche which helps in regeneration of neurons and glia [16]. This is substantiated by the extent of necrosis closely following the 40 Gy isodose distributions. For 40 Gy/25 in five weeks, the equivalent dose of 2 Gy (EQD2) is 36 Gy_3 _or 36.8 Gy_2_ with a BED of 61.3 Gy_3 _or 72 Gy_2_. Most of the brain parenchyma that underwent necrosis received 50 Gy/25# (EQD2:50Gy, BED: 100 Gy_2 _or 83.3 Gy_3_) and, hence, comparisons in the table have also been made with this in perspective. 

**Figure 5:**
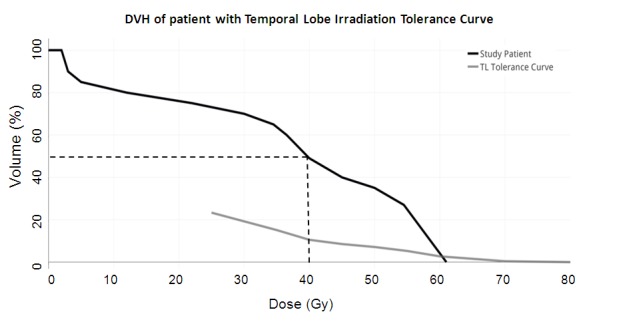
Dose volume histogram (DVH) of our study patient overlaid with the temporal lobe irradiation Tolerance curve By Sun, et al. [15]. DVH of our patient shows that 50% of the temporal lobe received ≤ 40 Gy, whereas tolerance curve suggests restricting the volume of temporal lobes receiving doses higher than 40 Gy to < 10%.

Our plan met the dose constraints provided by most authors, except Sheline, et al. and Su, et al. Recommendations made by Su, et al. are based on dosimetry from nasopharyngeal carcinoma series and, hence, may be impractical to adhere to when treating primary brain tumors [11]. However, they can be referred to when treating extracranial cancers. Figure 5 shows that the temporal lobe dose volume histogram (DVH) of our patient was significantly different than the newer guide suggested by Sun, et al. [15]. It is recommended that more conformal forms of therapy like intensity-modulated radiotherapy (IMRT) be utilized to achieve the strict dose constraints suggested by various authors in literature. Treatment with arcs (volumetric modulated arc therapy/ tomotherapy) would have resulted in better sparing of underlying brain parenchyma for superficial tumors like that of our patient’s.

## Conclusions

In the case presented above, TLN was incidentally diagnosed. The abnormality on the scan led to a detailed central-nervous-system-specific enquiry of symptoms. A detailed history with directed neurological examination may aid in earlier detection. It is important to understand that TLN may occur at lower doses than suggested and in treatment of other cancers than those frequently associated with it. The dose recommendations do not take into consideration the effect of concurrent chemotherapy on radiosensitivity of the brain parenchyma. Our patient was fortunate to have mild symptoms not significantly impacting his quality of life. However, there are many who tend to develop severe symptoms and in such cases early diagnosis and treatment of TLN may prevent clinical progression. Restricting doses to brain to as low as reasonably achievable is the key to preventing TLN.
